# Trust and Relationship Development Among Users in Dark Web Child Sexual Exploitation and Abuse Networks: A Literature Review From a Psychological and Criminological Perspective

**DOI:** 10.1177/15248380211057274

**Published:** 2021-12-07

**Authors:** Juliane A. Kloess, Madeleine van der Bruggen

**Affiliations:** 1 Centre for Applied Psychology, School of Psychology, 1724 University of Birmingham, Birmingham, UK; 2 The National Rapporteur on Trafficking in Human Beings and Sexual Violence Against Children, 4496 Leiden University, The Hague, Netherlands

**Keywords:** Dark Web networks, child sexual exploitation and abuse, online communication, trust, relationship development

## Abstract

The increased potential and speed of the Internet has changed the nature of sexual crimes against children. It enables individuals with a sexual interest in children to meet, interact, and engage in illegal activities. The literature review presented here aims to provide an overview of the current knowledge and understanding of trust and relationship development among users of online networks that are dedicated to the sexual exploitation and abuse of children. A systematic search using six databases was conducted to identify relevant literature from a psychological and a criminological perspective. Twenty-one studies met the inclusion criteria that centered around the key aspects of the literature review’s research question, namely, (i) child sexual exploitation and abuse, (ii) Dark Web platforms, (iii) online forums and networks, and (iv) trust and relationship development. Our findings reveal that the engagement in interpersonal communication and interactions with like-minded others serves various functions, including validation, normalization, and support, as well as access to expert advice, information, and material. Dark Web networks are high-stake and risky environments, where users have to manage a continuous flow of threats, with information about others and their trustworthiness being limited. The establishment and maintenance of trust is of social and technical relevance, and users have to navigate a number of demands and commitments. Findings are discussed in relation to theoretical and practical implications, as well as directions for future research.

Child sexual exploitation and abuse (CSEA) has existed long before the emergence of the Internet; however, new opportunities for offending are afforded by the online environment and its Triple A Engine, namely, anonymity, accessibility, and affordability (Cooper, 1998). This makes the Internet an attractive environment to seek out and pursue certain types of information and material, as well as engaging in various activities, while at the same time keeping one’s identity and participation hidden. It also enables individuals to connect with a large number of users without the restrictions of geographical proximity, and existing social networks (Leukfeldt et al., 2017). While the Surface Web allows individuals to adopt online identities or personas that are difficult to verify, the Dark Web offers additional “protection” by facilitating “near-complete anonymity” (Chiang, 2020) through its extra layers of encryption. More specifically, the Dark Web refers to a “part of the World Wide Web that can only be accessed using special software, such as The Onion Router (TOR), Freenet and I2P. It contains content that cannot be indexed by traditional search engines and provides anonymity for users and website operators” (National Crime Agency, 2019b, p. 6).

The Dark Web is a space where users can find anything from illegal drugs to stolen identities, as well as child sexual exploitation material (CSEM), and has a reputation of catering to some of the most notorious interests and goods through various platforms, with users taking advantage of its privacy and obscurity (Ntrepid, 2019). As part of these online communities and markets, users share advice and information, as well as best practices and recommendations, often relating to privacy, security, and avoiding detection, which enables “newbies” to learn from those with substantial experience of operating on the Dark Web (Chiang, 2020).

For users who are interested in (i) trading and sharing CSEM for personal or commercial reasons, (ii) communicating with like-minded individuals who have a sexual interest in children, and (iii) maintaining and developing “online pedophilic networks” (Beech et al., 2008), the Internet presents an ideal environment for pursuing and getting involved in these activities. Previously, individuals faced constant challenges and great personal risk when attempting to access material of this nature in the physical world. The Internet now enables users to connect with like-minded individuals across the world to form communities that offer moral validation, social support, and instant access to a continuous flow of information and material (Westlake & Bouchard, 2016).

Until 2017, when an international law enforcement operation conducted by Taskforce Argos, a branch of the Queensland Police Service in Australia, highlighted the deployment of undercover police officers by law enforcement agencies in an attempt to proactively investigate sexual offenses against children, offending behavior that takes place on the Dark Web had received little attention. By the time law enforcement took one of the main forums dedicated to CSEA on the Dark Web offline, the site had attracted one million user registrations. According to The Guardian (2017), 3000–4000 individuals were active users, and around 100 of these regularly produced and shared CSEM with the community.^1^

In 2019, the National Crime Agency in the UK identified 181,000 individuals who were members of organized crime groups and operated on some of the most problematic sites on the Dark Web that were dedicated to CSEA. It is of note that this number merely includes users who are known to be engaging in offending behavior and therefore represents a conservative estimate. The agency’s National Strategic Assessment of Serious and Organised Crime (2019) revealed that there were nearly 2.9 million accounts registered on these sites worldwide, with 5% believed to be from individuals residing in the UK. More worryingly, the number of referrals of identified occurrences of online CSEA from industry to the agency has increased by 700% since 2012 (National Crime Agency, 2019a). The agency argues that the anonymity afforded by the Dark Web continues to attract individuals who engage in serious and organized crime, with TOR being the main access point to services on the Dark Web. Furthermore, an ongoing growth in the volume of criminal trade notifications on TOR-based platforms has been noted, with CSEA online remaining a high-volume offense, and recorded instances of offending behavior increasing across the UK (National Crime Agency, 2019a), including the amount of CSEM that is being distributed (Europol, 2019).

Among the conclusions derived from the threat assessment undertaken by Europol (2019) was that the Dark Web is a key enabler for the trading in a wide range of criminal products and services. Although government and law enforcement agencies, as well as industry, have been publishing relevant figures and rates that give an indication of the ever-increasing problem they are facing in terms of tackling the use of the Dark Web for illegal activities, relatively little is known about the nature and role of CSEA forums on the Dark Web that are frequented by a large number of users (Finklea, 2017). In fact, what characterizes these forums is the enormous difficulty in accessing them for research purposes due to their illegal nature, and studies that specifically examine them (and other types of Dark Web forums) are therefore scarce.

Most studies that have been conducted from a psychological perspective have predominantly examined offenders’ characteristics and demographics, their motivations, and psychological variables, as well as conviction and reoffending rates, with a particular focus on individuals who view, download, distribute, or produce CSEM, and therefore access relevant platforms for these purposes, on the Surface Web. Some may be primarily motivated to access and download CSEM for the purpose of sexual stimulation, arousal, and gratification, whereas others may be driven to complete series of images and build a collection (Quayle & Taylor, 2002; Rimer, 2019). Again, others may be motivated by the financial gain associated with dealing with this type of material.

The few studies that have looked at the nature and role of online communities and networks, geared toward individuals with a sexual interest in children, have found that their organizational structures are similar to pedophile rings and other criminal networks in the physical world, and that they provide users with a space that serves the function of social and peer support, validation, and access to expertise. More specifically, the online environment provides users with opportunities to access a wide range of information and resources, as well as corresponding and interacting with like-minded individuals. Groups of individuals form communities online which act to validate users’ attitudes and beliefs, as well as their sexual interests, preferences and behaviors. Something that keeps them attractive, and suggests popularity, is the increasing membership, and the fact that these platforms are uncensored and peer-moderated spaces, enabling users to interact freely without constraints and sanctions. These groups or communities thereby take on the role of a support mechanism or system for individuals who have a sexual interest in children, which is absent in their lives in the physical world (Holt et al., 2010; Martellozzo, 2015).

Studies from a criminological perspective have emerged that specifically explore group dynamics, such as the development of trust, in cybercriminal networks on the Dark Web, including those geared toward hackers and marketplaces where illegal goods (e.g., drugs and weapons) are exchanged. A small number of these studies also focus, in part, on networks that are dedicated to CSEA. The question arises as to how cooperation and trust (defined as a mechanism to “cope with risk and uncertainty in interactions with others”; (Von Lampe & Johansen, 2004, p. 103) between co-offenders is established under conditions where users do not know each other’s true identity, where no regulatory body is present to enforce rules, and where trust may, therefore, be easily betrayed (Lusthaus, 2012). Here, criminological studies have reported similar findings to those of a psychological nature, emphasizing the role of virtual communities in normalizing and justifying sexual relationships with children, and encouraging users to engage in this type of offending behavior (Cohen-Almagor, 2013; Holt et al., 2010).

Overall, the aim of the present review is, therefore, to provide an overview of the current knowledge and understanding around the nature of trust development in online networks, and how relationships are formed among members of these, both from a psychological and a criminological perspective, in order to derive insights that may help explain and make better sense of the way users on CSEA forums on the Dark Web communicate and interact with one another. Given the varied focus of the disciplines of psychology and criminology, we thereby hope to offer a more comprehensive overview by reviewing relevant literature from two perspectives.

## Method

The literature review presented here employed a systematic search strategy in order to identify any articles that were of relevance to answering the research question. We were predominantly interested in the development of trust and relationships among users on Dark Web networks that are dedicated to CSEA. However, in light of the scarcity of existing studies, articles that explored aspects related thereto on platforms both on and off the Dark Web, and in relation to other cybercriminal activities, were still included. A number of different aspects were identified when reading and re-reading the 21 articles and are synthesized across the studies according to the perspective they represent, thereby offering an insight into the various processes that take place as part of interpersonal communication and interactions on different Internet communication platforms.

### Search Strategy

Six databases, including Google Scholar, JSTOR, ProQuest, PsycNET, Scopus, and Web of Science, were searched between June 2019 and August 2019 using a combination of different search terms. The search terms centered around the key aspects of the literature review’s research question, namely, (i) CSEA, (ii) Dark Web platforms, (iii) online forums and networks, and (iv) trust and relationship development. For databases that allow the filtering of results, the search was limited to the topic areas of psychology, crime, sociology, and social science(s).

### Search Results

**Figure 1.**
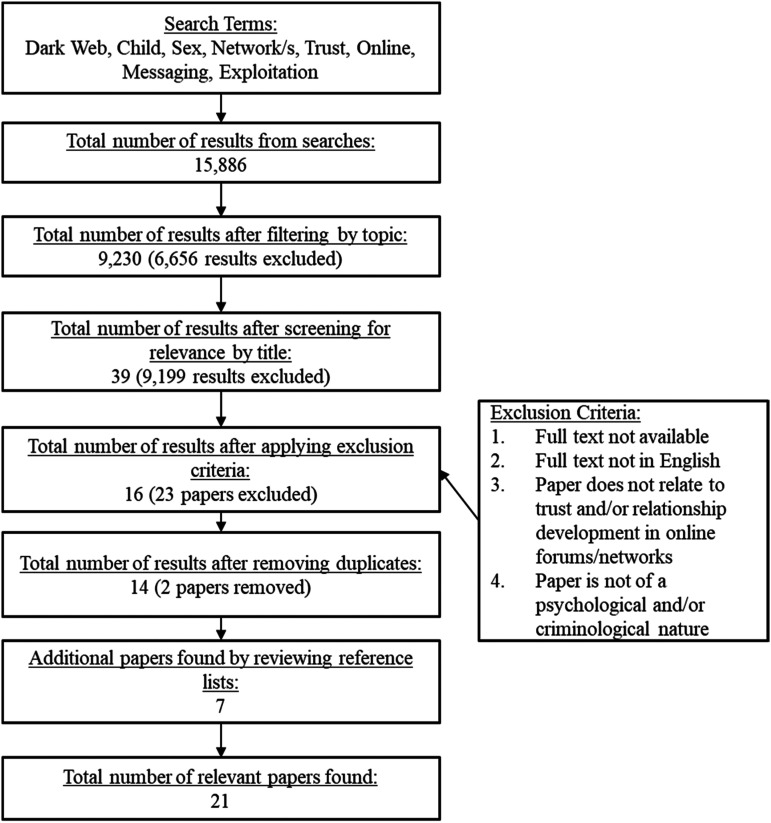
PRISMA flow diagram of the systematic literature search.

A total of 15,886 articles were returned by using several combinations of the search terms. Following the application of filters by topic area (within the databases that allowed this), the number of total articles reduced to 9230. The titles of these articles were reviewed for relevance, resulting in a further reduction to 39 articles. Once our exclusion criteria were applied, and duplicates were removed, 14 articles remained. Finally, the reference lists of the 14 articles were reviewed, and a further seven articles were identified. This resulted in a final set of 21 articles. Figure 1 presents an overview of the steps that were undertaken to achieve the final set of articles included in the review.

### Study Characteristics

#### Psychological Articles

Of the 21 articles, eight contained a psychological or anthropological focus. Of these, three were conducted in the US and five were conducted in the UK. All except one of the articles were studies that examined qualitative data derived from Internet communication platforms, semi-structured interviews, or in-depth ethnography (with the one exception drawing on a range of data from two empirical studies). Two of the eight articles described a thematic and content analysis of a “boy love” support forum, with one article (UK) involving the revisiting of the original study (US). The other two articles from the US examined (i) posts on an Internet message board and (ii) threads from web forums, both geared toward individuals with a sexual interest in children. Of the remaining four articles from the UK, one presented a detailed overview of policing online child sexual abuse by drawing on findings from two empirical studies that were conducted between 2003 and 2013, and three involved semi-structured interviews with individuals who had been arrested for or convicted of offenses related to CSEM (*n* = 7 + 13 + 31 = 51). In addition to semi-structured interviews, one study also employed an ethnographic approach that involved 17 months of participant observation (*n* = 81) in a UK group program for individuals who had been arrested for offenses related to CSEM. Table 1 provides an overview of the study characteristics of the psychological articles, including study aims, participant details, methodology, and main findings.

**Table 1.** Overview of Study Characteristics and Main Findings from Psychological Articles.

Study	Aims	Participants	Method and Data Analysis	Main Findings
Durkin and Bryant (1999)	To investigate how pedophiles who use the Internet account for their deviance.	41 self-identified pedophiles who participated in an online forum.	93 posts by 41 self-identified pedophiles were analyzed using content analysis to determine the presence or absence of a number of different variables: (i) Account offered, (ii) condemnation of condemners, (iii) denial of injury, (iv) claim of benefit, (v) appeal to loyalties, (vi) BIRGing, and (vii) polythematic account.	Slightly more than half of the participants offered some type of account in defense of pedophilia or adults engaging in sexual activity with children: (i) Denial of injury = 39%, (i) condemnation of condemners = 31.7%, (iii) polythematic account = 24.4%, (iv) BIRGing = 14.6%, (v) claim of benefit = 9.8%, and (vi) appeal to loyalties = 4.9%.
Holt et al. (2010)	To investigate the cultural norms that govern pedophile communities, and how individuals become encultured in these online communities.	Five forums run for and by pedophiles with 198, 40, 224, 123 and 418 users.	705 threads from the five forums were analyzed using inductive grounded theory to draw normative orders which are sets of rules and practices oriented to a common value. The forums were identified and selected through a snowball sampling procedure.	Members felt isolated and adopted language emphasizing the separation between them and the outside world. Forums therefore provided a sense of belonging. Members also often discussed sexuality, including fantasies, past experiences or sexual preferences that involved children, and presented with knowledge around legislation and recent policing activity. Members also advised each other on issues of security and how to avoid detection, and offered advice on approaching children online and offline.
Malesky and Ennis (2004)	To understand the specific functions of message boards for individuals who make use of them and to investigate the type of cognitive distortions evident in forum posts.	An unknown number of male members of a “boy love” forum.	234 posts over seven days were analyzed using a cognitive distortion checklist with 11 separate categories representing justifications, misperceptions of consequences, attribution of blame to the victim, and supplemental distortions.	27% of posts contained at least one distortion, with euphemistic labeling being the most common distortion and justification (present in 24% of posts). All other distortions were only present in 2–5% of posts. Over 20% of posters were seeking validation of their beliefs, while over 50% provided material such as images and poems relating to boy love. Over 60% of posts contained social elements, with over 50% of posts serving multiple purposes.
Martellozzo (2015)	To contribute to the field of policing online child sexual abuse, and the development of global police practice.	21 officers and forensic examiners.	Online transcripts between offenders and undercover officers, as well as observational notes and interview transcripts from semi-structured interviews, were analyzed using grounded theory to identify and develop themes.	Several themes were identified in relation to (i) the difficulties faced by officers posing as adults with a sexual interest in children and children; (ii) the immersion required to remain adaptable and convincing; and (iii) the community aspect of child sexual abuse forums, with particular reference to a forum where trust was built via engagement and following set rules.
O’Halloran and Quayle (2010)	To revisit the forum investigated by Durkin and Bryant (1999), and assess how the forum’s nature had changed over 10 years.	23 contributors to the “boy love” forum with membership ranging from several months to 10 years.	127 posts by 23 self-identified pedophiles were analyzed using content analysis (using the same template as Durkin and Bryant (1999)).	The most common justification was the condemnation of the condemners, followed by denial of injury, claim of benefit, denial of victim, appeals to higher loyalties, and basking in reflected glory. Members who shared their justifications appeared to do so to alleviate feelings of guilt, referring to the forum as a kind of learning environment.
Quayle and Taylor (2002)	To investigate the relationship between those who are sexually interested in children and the Internet.	13 men convicted of downloading or distributing child sexual exploitation material.	13 semi-structured interviews were analyzed using a discursive framework by focusing on the function of interviewees’ accounts. Data were subsequently organized into emerging categories based on similarities and differences.	Child sexual exploitation material served the function of sexual stimulation, collecting behavior, escaping real life, and therapy. Only some interviewees used child sexual exploitation material as a method for facilitating social relationships. Trading images via instant messaging enabled the formation of friendships and built networks based on trust. The Internet offered a way of creating a private and arousing world.
Rimer (2019)	To examine child sexual exploitation material users’ constructions of children, and childhood online and offline, and explore how these factor in their offending.	81 men arrested for viewing and possessing child sexual exploitation material, of which 31 took part in semi-structured interviews.	31 of the sample took part in semi-structured interviews, which employed a 25-question guide split into five sections: background, Internet and pornography, children and childhood, and current circumstances. The transcripts and field notes were analyzed using thematic analysis in order to identify themes.	Four themes were identified: (i) constructions of children in the offline world; (ii) constructions of children in the online world; (iii) negotiating “realness”: making children anonymous, distant and other; and (iv) negotiating “realness”: victim empathy. Participants were found to construct a fundamental difference between children online and in the physical world, with children in images not being perceived as “real” and sexualized. This facilitated offending in terms of overcoming barriers, and allowing participants to hold conventional beliefs about children while engaging in incongruent online activity.
Winder and Gough (2010)	To understand child sexual exploitation material offenders, and how they rationalize and defend their offending behavior.	Seven convicted child sexual exploitation material users/distributers aged between 20 and 60.	Seven semi-structured interviews were analyzed using Interpretative Phenomenological Analysis. The interview guide centered around family environments, past offenses and feelings about them, the impact of offenders’ actions on other areas of their life, other offenses and offender identity, treatment, as well as other individuals committing sexual offenses, and future plans outside prison.	Several key themes were identified in relation to (i) obsession and compulsion, (ii) isolation, (iii) escapism, (iv) enjoyment, and (v) self-distancing. Most individuals sought to distance themselves from contact sexual offenses/offenders. Individuals also sought to minimize the impact of their actions by construing the offense as an imaginary affair. Following treatment, individuals reported greater accountability for their actions.

### Criminological Articles

Of the 21 articles, 13 contained a criminological focus. Of these, one was conducted in the US, one in Austria, one in Taiwan, two in Australia, and four in Canada and the UK. The majority of the studies (*n* = 8) used naturally occurring, real-world data for the purpose of analysis, including users’ forum messages and posts, as well as information about their status, reputation scores, and ratings. Three of these studies used a qualitative approach to data analysis (with one article combining this approach with social network analysis). One study used a quantitative approach, and three used a combination of both. Six studies explored forum sustainability, the distribution and development of user reputation, social and market dynamics on a forum, and trust in general. One study adopted the Event Analysis of Systemic Teamwork (EAST) method, an “integrated suite of methods for analyzing performance and behavior in human-technical systems” (Lacey & Salmon, 2015, p. 121), to investigate the tasks and interactions undertaken by first-time enrollers in illicit markets, as well as trust establishment more broadly. A further two studies conducted interviews with professional experts to establish how users with an interest in CSEA use the Internet more generally, and how mechanisms of trust and distrust are addressed in online networks specifically. One other study conducted interviews with drug users and vendors who were active on a specific Dark Web forum in order to examine the similarities and differences between drug dealing on Dark Web markets and drug dealing in the physical world. The interview-based studies used content and thematic analysis for the purpose of analyzing their data. Finally, two articles were literature reviews. Table 2 provides an overview of the study characteristics of the criminological articles, including study aims, participant details, methodology, and main findings.

**Table 2.** Overview of Study Characteristics and Main Findings From Criminological Articles.

Study	Aims	Participants	Method and Data Analysis	Main Findings
Afroz, Garg, McCoy, & Greenstadt (2013)	To investigate what makes a particular forum sustainable, and what distinguishes sustainable forums from those that fail.	Five cybercriminal forums, each of between 8,000 and 19,000 users.	An economic framework was applied to examine forum sustainability. The framework considered a number of factors: (i) the monitoring of forum members, (ii) the rate of change of members and their connectivity per month, (iii) the social connections and communication between members, (iv) exclusion criteria, (v) forum enforcement, and (vi) member punishment.	The use of a cheap monitoring system was associated with greater sustainability. This enables the identification of non-trustworthy forum members. In addition, forum growth and connectivity should be consistent and moderate. Less successful forums exhibited staggered growth, with bursts of growth and sudden declines in connectivity. Forums exhibiting a gradual increase in communication were sustainable in light of the growth in social capital and trust building. Forums that use enforcement were also associated with higher sustainability.
Broadhurst et al. (2014)	To review a variety of cybercrime organizations, and explore the nature of groups engaged in cybercrime.	N/A	The existing literature was reviewed and critically evaluated, drawing on cybercrime typologies, and making reference to existing cybercrime cases, offenders and operations.	A wide variety of organizational structures are involved in cybercrime. The organizations referenced focused on goals, such as freedom of information, defiance of authority, sexual gratification, and technological prowess. A members-only online CSEA group is described to illustrate an example of cybercrime and its offenders.
Cohen-Almagor (2013)	To understand how child sexual offenders use the Internet, and how to counteract them.	14 Internet experts and senior law enforcement officers in various countries were consulted.	A critical reflection of books, academic papers, news articles, as well as government and law enforcement reports, was conducted, combined with a total of 11 expert interviews.	The Internet has enabled the emergence of online communities for the purpose of exchanging information, seeking social support, and meeting desires. The social connections and relationships that are formed encourage trust development. Moreover, sexual interest in children is normalized and justified. Collaboration across various parties is required in order to reduce the threat posed by child sexual offenders.
Décary-Hétu and Dupont (2013)	To examine how reputation is distributed amongst a single network of botnet hackers, and how it relates to criminal achievement.	One forum with 20,270 members who posted 248,634 public messages was selected and analyzed from February 2007 to November 2011.	A custom program was used to download members’ account information, posts, reputation scores, and awards received from administrators. A content analysis was conducted on a subset of messages, and a multi-level predictive model was used to establish how reputation was distributed among individual members.	The model showed that reputation significantly correlated with the number of awards received, time spent on the forum, and size of a member’s ego network. Nurturing positive roles and openness led to more sustained levels of reputation, as did engagement in social networking, and helped to reduce mistrust.
Dupont (2013)	To explore the norms and practices that govern the interactions between malicious members online.	113 chat logs between 10 male botnet hackers, who were aged between 17 and 25 years, and any other user, in which malicious hacking was mentioned.	A qualitative data analysis software (QDA Miner) was used to facilitate the coding, annotating and retrieving of the dataset. The data was further analyzed using social network analysis.	Some members were more active than others; however, this was not indicative of social skills or trust. Arguments were also frequent among members. High-trust members often exchanged pieces of code, servers, login details, and warnings. These members also discussed personal issues. Low-trust members shared information, but only to a certain extent, and were more often characterized by failures and ridicule, fostering distrust in the forum.
Dupont et al. (2017)	To explore the social and market dynamics of Darkode, an invite-only cybercrime/hacking forum.	A dataset of 4,788 screenshot files from the forum’s discussion threads covering data between 2009 and 2013.	The selection process, through which 344 potential new members introduced themselves to the community in order to be accepted into the forum, was examined using a qualitative approach in order to determine whether a rigorous procedure significantly enhanced trust and contributed to the efficiency of the marketplace.	Despite the security-minded reputation of the forum, many members were let in for profit. Trust and reliability therefore remained elusive, and interactions were often fraught with suspicion and accusations. It was concluded that high-end markets also face distrust.
Dupont et al. (2016)	To investigate how trust (as measured by a reputation system) is distributed in quality and quantity between members, and how it fluctuates over time.	A large hacking discussion forum of 29,985 hackers (20,768 general hackers, and 9,127 botnet hackers) who had been rated by 9,177 other members through 449,478 different events, over a 27-month period.	A qualitative analysis was conducted of 25,000 feedback comments in order to examine whether they were a positive or negative evaluation on (i) the business relationship, (ii) someone’s contribution, (iii) an assessment of an individual’s interaction with the feedback provider, (iv) their technical ability, (v) humorous, sarcastic or absurd comments on a member’s actions or skills, and (vi) unreadable or meaningless comments.	The variation in positive feedback was attributed to the different natures of the groups, with botnet hackers being involved in a market-based community where members have more to lose. There was a bias toward reporting positive outcomes which reduced the utility of the ratings, as did the low participation rate in the rating system. The qualitative analysis revealed that trustworthiness was mostly grounded in humor and sarcasm.
Hsu et al. (2011)	To examine the antecedents of trust in virtual communities.	324 members of tech-related virtual communities, 244 of which were male, and 80 of which were female.	Surveys were distributed, including questions on knowledge growth, perceived responsiveness, social interaction ties, shared vision, system quality, knowledge quality, trust in the system, and knowledge-sharing intentions. A research model was tested, using structural equation modeling, to examine the influence of each factor on other connected ones.	Shared vision, perceived responsiveness and knowledge growth had significant effects on trust in members, while knowledge quality had a significant effect on trust in the system. Moreover, trust in the system and its members significantly affected members’ knowledge-sharing intentions.
Lacey and Salmon (2015)	To investigate the tasks and interactions undertaken by first-time enrollers on illicit markets, and trust establishment between them and other members.	The Republic of Lampeduza (a carding forum) with over 4,000 trusted members and over 71,000 posts.	Event Analysis of Systemic Teamwork (EAST) was used to describe the goals and future tasks performed by members of a system, the organization, and communications between members, as well as how information is distributed across members in a system. The researchers conducted a hierarchical task analysis (HTA) to break down the enrollment process into smaller goals and plans, and observed all steps involved in registering and enrolling.	The HTA revealed three key steps with multiple sub-steps. A key trust-building step was the allocation of an unknown reviewer who specifies the trust-building requirements. The communication and organization analysis revealed a complex network involving non-human and human components. The information network analysis indicated that quality assurance is of high importance in trust-building strategies.
Lusthaus (2012)	To investigate the mechanisms by which cybercriminals address the issue of distrust in online networks.	Nine Internet security practitioners, law enforcement officers, and cybercriminals.	Interview data were considered alongside legal documents, security firm reports, and media articles. All interviews were audio-recorded, transcribed and analyzed using data were thematic analysis.	Prospective cybercriminals must present a criminal identity as a key step in trust development. These identities can be tested via background checks, criminal acts, and information hostages. To assess the attributes of a prospective cybercriminal, existing forums use criminal displays, referrals, and expertise demonstrations. Enforcement measures are also employed.
Nurse and Bada (2018)	To examine the group dynamics of cybercrime forums from several perspectives.	N/A	The existing literature was reviewed and critically evaluated, drawing on online platforms used by cybercriminals, the types of groups present (including their motivations and actions), and how these groups form and operate.	Cybercriminal groups have different structures and goals. Trust in online groups is characterized by the integrity of the system to maintain anonymity, and can be directed to the community, information sources, potential partners, and authorities. Many forums use screening measures to test trustworthiness of members. Individuals have to strike a balance between changing nicknames in order to distance themselves from past crimes, and revealing certain aspects of their identity for the purpose of building a reputation and attracting criminal collaborations.
Tzanetakis et al. (2016)	To examine the similarities and differences between drug dealing offline and on Dark Web markets in terms of violence, trust, and the logistics of distribution.	214 “conventional” drug users and/or dealers and four vendors on the online market Agora.	Participants took part in interviews as part of an existing mixed-method research project. Qualitative case studies were conducted with the Dark Web vendors, considering data such as customer feedback, profile pages, and forum chat material, and were analyzed using content analysis.	Trust in online networks was proactively promoted in order to increase cooperation and sales, and attract new customers. Vendors’ ratings and conflict resolutions help to build a trustworthy reputation. Trust is therefore characterized by network structure and good conduct. In offline communities, trust was more based on interpersonal relationships. Third-party conflict resolution was more common in online communities.
Yip et al. (2013)	To examine the structure of organized cybercrime, and explore the facilitation of trust using theories derived from social psychology, organized crime, and transaction cost economics.	Data from public discussions on online carding forums.	Theories of transaction cost economics were applied to data from shut-down online markets and carding forums. Uncertainty was treated as a cost to the transactions and was therefore used as the unit of analysis, in order to examine the mechanisms cybercriminals use to control two-key sources of uncertainty: (i) the quality of merchandise, and (ii) the identity of the trader.	To mitigate uncertainty and facilitate trust, discussion forums adopt a hybrid organizational structure. Quality assurance via reputation systems was one method used to increase institutional trust. However, social networking was the main means through which interpersonal trust between members was increased. Through exchanges, members learn good conduct and group rules, and shared personal information and feelings toward their profession and any associated risks. Having a forum built on interpersonal and institutional trust is therefore imperative for success, as this reduces transactional cost and allows forums to grow.

## Results and Discussion

### Marginalization and Semantic Manipulation

In the study by Holt et al. (2010), users clearly recognized that their sexual interests and preferences were different from the wider population, which carries with it marginalization and social stigma. Some described fearing for their personal safety and being persecuted. Within this context, users sought to distinguish between individuals who engage in various sexual behaviors involving children. More specifically, there is a group of users who proclaim that they love children and would never hurt them (often referred to online as “child lovers”); there is another group of users who are open about engaging in the sexual abuse of children (often referred to as “pedophiles”). The former actively attempt to distance themselves from the “pedophile” label and view themselves as different and not harming children. Semantic manipulation by means of differentiating between “child lovers” (whose attraction to children is portrayed as a romantic relationship) and “pedophiles” is clearly important for the former in terms of preserving a positive self-concept (Holt et al., 2010).

**Table 3.** Summary of Critical Findings.

	Critical Findings
Psychological Literature	- Online communities and networks serve various functions, including validation and support, as well as advice, guidance, and information- Users of online platforms appear to meet social and interpersonal needs, including connecting and building relationships with others
Criminological Literature	- The establishment of trust and relationships is accomplished through social and technical mechanisms and is directly related to forum users’ online identity- Once trust is established, it is maintained through the social aspects of dedication to the community

**Table 4.** Summary of Implications for Practice, Policy, and Research.

	Implications
Practice	- Users’ motivation for accessing online platforms is predominantly of a sexual nature- Users with a sexual interest in children experience marginalization and social stigma, sometimes fearing for their personal safety
Policy	- Law enforcement having to meet increasingly more difficult to achieve criteria to join particular networks- Knowledge around how users operate benefits law enforcement for the purpose of early detection, and informing operational strategies
Research	- To better understand individuals who interact on Dark Web networks that are geared toward the sexual exploitation and abuse of children-To explore the role of online networks in the escalation of offending behavior

### Justifications for Engaging in Offending Behavior Against Children

Malesky and Ennis (2004) analyzed users’ posts on an Internet message board with a particular focus on distorted thinking that was supportive of offending behavior involving the sexual abuse of children. The term “cognitive distortions” is often used in the literature to refer to a very broad range of both post-offense explanations, and cognitive processes during offending, including excuses, rationalizations, beliefs, perceptions, justifications, denials, minimizations, and defenses (Maruna & Mann, 2006; ÓCiardha & Ward, 2013). Firstly, commonly held attitudes and beliefs that functioned to strengthen users’ attempts at building a credible argument in defense of pedophilia (i.e., a sexual interest in children) related to (i) denial of injury to children, (ii) denial of victim, (iii) claim of benefit, and (iv) condemning the condemners (i.e., discrediting others who challenge them) (O’Halloran & Quayle, 2010). The dominance of justifications over excuses in O’Halloran and Quayle’s (2010) study suggests that users did not consider that sexual contact with children was wrong, but merely that it is viewed negatively by wider society. The sharing of these justifications was found to be an important part of discussions that featured on the support forum the authors analyzed.

Secondly, participants in Quayle and Taylor’s (2002) and Winder and Gough’s (2010) studies claimed that accessing CSEM for the purpose of sexual stimulation, arousal, and gratification, often accompanied by masturbatory activity, acted as (i) therapy for dealing with negative emotional states, such as loneliness, depression or relationship breakdowns; (ii) a substitute for committing contact sexual offenses in the physical world; and (iii) a safe outlet for feelings that would otherwise lead to a contact sexual offense. For others, it arguably acted as a blueprint and stimulus for a contact sexual offense, while some participants maintained that preventing masturbation was “accentuating the problem by provoking more contact offenses” (Quayle & Taylor, 2002, p. 131).

Further justifications commonly reported in the studies that involved interviews with individuals who were convicted of offenses related to CSEM include (i) that it is “just pictures” (Rimer, 2019); (ii) drawing comparisons between image offenses and contact sexual offenses (e.g., “I am just looking,” “nobody is getting harmed”; Rimer, 2019; Winder & Gough, 2010, p. 130); (iii) referring to children in the images as being happy and smiling; (iv) experiencing sexual abuse themselves; (v) citing legislation in other countries, where age of consent is such that it legalizes the sexual behavior engaged in by the individual; and (vi) claiming that the production of imagery offers employment for children in particularly poor parts of the world, without which children would starve (Quayle & Taylor, 2002; Rimer, 2019; Winder & Gough, 2010). In addition, participants in the Rimer (2019) study constructed children depicted in CSEM as sexualized and less or not real, which is partly facilitated by the anonymous nature of the online environment (and the fact that they are unknown to them). This therefore allows individuals who view this type of material to objectify children, and become desensitized, detached, and distanced to the content, which assists in the overcoming of barriers, and enables the continued engagement in offending behavior (Rimer, 2019).

Participants also commonly described the process of accessing CSEM as addictive and compulsive. This not only implies a loss of personal agency, but also allows users to present their behavior as out of their control (e.g., “I can’t help myself”), absolving them of culpability (Quayle & Taylor, 2002). Similarly, participants in Winder and Gough’s (2010) study presented their offending in the context of being driven by obsessions and compulsions, thereby elevating “the role of psychological illness over personal choice and culpability, while isolation privileges a situational over an individual explanation” (p. 128–129). Through talking about CSEM by highlighting its addictive and compulsive properties, it serves to distance the user from the material and both minimizes and removes any personal responsibility.

Overall, this is largely facilitated by the perceived non-contact nature of offenses related to CSEM, with images being described as “mundane and innocuous” (Winder & Gough, 2010, p. 129) to negate their severity. Participants further claimed that children smiling in images indicated that they were happy and that victims who were not aware of being recorded were not harmed. Furthermore, they distanced themselves from the label and identity of “sexual offender,” denying that they were any danger to children, and presenting offenses related to CSEM as less wrong and harmful than contact sexual offenses. Another attempt to justify their offending behavior was by means of the “looking-but-not-touching” mitigation, implying no knowledge of or contact with the child depicted in the material (Rimer, 2019; Winder & Gough, 2010).

Participants who referred to the accessing and downloading of CSEM as the main motivator for using Internet communication platforms made little to no reference to the fact that this material depicts vulnerable children, but rather drew comparisons with other commodities that are known for collecting behavior (e.g., stamps). Collecting further facilitates the objectification of children, given that images in this context are treated as currency (Quayle & Taylor, 2002). It is of note that those who were engaging in this type of behavior not only collected CSEM but also other forms of pornography, despite presenting with a sexual interest in children. Others described the progression and escalation from legal adult pornography to seeking out more novel and extreme material (i.e., CSEM) (Quayle & Taylor, 2002; Rimer, 2019).

### Function of Online Communities and Networks

Given that individuals with a sexual interest in children represent a marginalized group, online communities serve the function of offering support by, and understanding from, like-minded individuals in various ways (Holt et al., 2010; Martellozzo, 2015). Holt et al. (2010) considered the role of such communities in developing a so-called “subculture” of “pedophiles,” as part of which attitudes, beliefs, and justifications are fostered that support relationships with children. The authors concluded that “prominence placed on marginalization may act as a primer in individuals’ behavioral chain, freeing them to offend as they are already social outcasts” (Holt et al., 2010, p. 21). Furthermore, through facilitating connection with like-minded individuals, online communities create an environment in which individuals’ attitudes, beliefs and behaviors are normalized, validated, and even minimized. This is particularly powerful for those who are seeking to come to understand their sexual interests of or attraction to children (Martellozzo, 2015), and ultimately achieves social cohesion and a sense of belonging among individuals (Holt et al., 2010). In their study of an online discussion group, Holt et al. (2010) found that many of the conversations that took place on it resembled daily catchups, with users telling each other about their day and what they were up to. Furthermore, according to Ward and Hudson (2000a), offenders with a sexual interest in children gravitate toward environments with like-minded individuals who hold similar attitudes and beliefs that support their lifestyle and belief system.

More specifically, the largest percentage of posts (63%) in an analysis of an Internet message board fell into the category of communications that were social in nature and did not specifically involve content related to “boy love” (Malesky & Ennis, 2004). In addition, slightly more than one-fifth of the posts were classified as validating pedophilic beliefs and relationships. One may argue that users did not feel compelled to defend their beliefs to themselves or others through cognitive distortions, and felt relatively accepted in the community, which may be expected given that it was geared toward users with an interest in “boy love,” and likely attracted like-minded individuals. The authors concluded that users may find a sense of membership and community through participation in and interaction on platforms online, including connecting with others and building relationships. Especially for those who are potentially marginalized in their communities, or society more broadly, this may lead to feelings of empowerment. Seeking out and joining online communities therefore clearly serves the function of social connection, particularly where this is absent and missing in someone’s personal life in the physical world. In addition, online networks may also provide an opportunity for users to excel at something, achieve a particular status in the community, and gain the respect of others. This is especially powerful where users’ identities in the physical world bear little resemblance to the identity they have created online. In particular, users described creating a secret and separate world to their reality, which for some took on the role of a fantasy in comparison to their mundane everyday life. The element of danger and illegality in such cases acted as an excitement and escape (O’Halloran & Quayle, 2010).

In addition to meeting the more social and interpersonal needs of users, online communities geared toward individuals with a sexual interest in children also serve the purpose of facilitating access to advice, guidance, and information. This may be related to (i) approaching children (both online and in the physical world), (ii) initiating and developing friendships with children, (iii) gaining access to potential victims (for sexual abuse in the physical world, including the production of CSEM), and (iv) sharing “best practice” and “what works” with regard to all of these, as well as achieving compliance in victims, ensuring non-disclosure, concealing one’s identity, and avoiding detection overall (Holt et al., 2010; Martellozzo, 2015).

To practice security and avoid detection, much of the content of the conversations in Holt et al.’s (2010) study focused on advice around carefully managing personal information and activities, as well as being mindful of the level and type of information contained within posts, and where these are posted. For example, some users may present their experiences as dreams or fantasies (rather than actual acts or activities they engaged in or happened). Other advice would center around privacy issues and technical requirements in order to protect users’ true identities and keep their equipment secure. Restrictive guidelines around what content was allowed to be posted or published helped to minimize negative attention for the platform, including in relation to the exchange of illegal material. Throughout, the conversations were accompanied by users’ concerns around safety and the law, clarifying definitions of what is legal and illegal. They further expressed their opinions about what they perceive to be harmful to children (or not), and whether CSEM represents contact sexual offending, debated about whether children are able to consent, and discussed recent cases, arrests, and prosecutions of individuals involved in CSEM (Holt et al., 2010).

Finally, online communities follow established group dynamics and hierarchies of status, expertise, and apprenticeship (O’Halloran & Quayle, 2010). According to Martellozzo (2015), some of these may be compared to organized criminal organizations, whereby members are required to present with certain personal qualities in order to gain membership status (such as honesty, honor, obedience, and participation). More specifically, “membership was reinforced by having material to trade, by behaving correctly, and by following the rules for trading. Once status had been achieved through membership of the group, trading reduced, and, instead, the social function of the online exchanges, and the ability to be on the inside and obtain special photographs, was more important” (Quayle & Taylor, 2002, p. 346). As noted previously, within online communities, imagery may therefore act as a medium for exchange, whereby users may look to build a large collection, complete a series of images, and thereby look for missing parts, as well as distributing new material, which ultimately contributes to their standing in the community (in terms of the nature of the material, its size, completeness, and value, with participants in Quayle and Taylor’s (2002) study referring to some images as “Picassos”).

The forming of social relationships and establishment of social cohesion among a group of users is further facilitated by this very exchanging and trading of imagery, which requires users to come into contact with others who are similarly interested in, and engage with, this type of material (Quayle & Taylor, 2002). The possession of imagery was often also a requirement for joining and becoming a member of a community or forum. Connections or friendships with certain users may also advance the status of another, while at the same time facilitating access to their collection and victims. Imagery was often described as currency that enabled the building of one’s reputation and trust with other users, contributing and helping to maintain relationships with them. In Quayle and Taylor’s (2002) study, participants also referred to the importance and prioritization of relationships over imagery, describing the community as something like a club, whereby users were provided with and given what they were looking for and wanted, while ensuring that the forum was running smoothly. The exchange of imagery was therefore contextualized as a commodity that enabled social cohesion.

Overall, being a member of one of these online communities creates a sense of belonging to an in-group, which works to establish elements of trust and “being in it together.” According to Martellozzo (2015), trust is further developed through engagement and following set rules. Members who otherwise feel oppressed by wider society in the physical world are immersed in a strong social network online, as part of which users may share their likes and dislikes, sexual interests and preferences, as well as their daily encounters or experiences. Holt et al. (2010) concluded that “by sharing information with others in an environment where feedback, reciprocity, and a congruence of opinion, can be found, the forum users are able to connect in ways that validate and support their actions” (p. 20).

Given the nature of online networks that center around CSEA, including its material, it is to be expected that a certain level of trust among users is established through mere association with and involvement therein. Being a member of an online community thereby gives users the impression of being part of an in-group, which works to reinforce one’s perception that they are “in it together,” and “them against us.” In fact, this raises important questions in terms of the power these online networks have in contributing to the escalation of offending behavior, both with regard to acceleration and aggravation (O’Halloran & Quayle, 2010); they provide access to expert advice, guidance, and information, including how to find victims or particular material, as well as detailed descriptions of various modi operandi, and how to avoid detection (Woodhams et al., 2021). Arguably, receiving support from like-minded individuals in the way it has been described here is suggested to promote pro-offending beliefs in socially isolated individuals (O’Halloran & Quayle, 2010). Interactions as part of online communities provide immediate positive reinforcement for users, and their narratives can take the form of either excuses or justifications that aim to minimize questionable activity.

It is important to highlight how difficult it is to verify users’ true intentions behind what they post (O’Halloran & Quayle, 2010); they may provide certain content to comply with rules and regulations, or to seek acceptance, recognition, and status within the network. In order for trust to be established and maintained, and for a platform to remain secure, users may merely be able to join a network and become a member by being invited or meeting specific criteria. This not only prevents law enforcement from infiltrating the network but also ensures that only users who are serious about becoming members join the community.

### Trust Establishment in Online and Offline Communities

Trust is established differently in online and offline environments. In offline environments, trust may be developed through previously existing ties, personal bonds, common interests, and values, as well as face-to-face encounters. However, these are largely lacking in online environments, and the challenge is therefore that trust has to be established under anonymity without knowledge of who one’s co-offender is, making it fragile and difficult to sustain (Décary-Hétu & Dupont, 2013; Dupont et al., 2016; Tzanetakis et al., 2016). Nevertheless, users have to trust both one another (to be willing to share information and co-offend), and the reliability of the (technological) system, including its standards and mechanisms (Hsu et al., 2011).

On the one hand, the anonymous nature of the online environment affords users protection against exposure and a place where they can engage in conversation and interaction, making it suitable for supply to meet demand and the exchange of technical expertise (Nurse & Bada, 2018). On the other hand, anonymity hinders the process of trust establishment and development (Dupont et al., 2016, 2017; Lusthaus, 2012). Those who engage in offending behavior online therefore have to carefully balance between masking their identity to avoid exposure and detection, and revealing elements of it for the purposes of criminal cooperation (Lusthaus, 2012; Nurse & Bada, 2018).

### Trust Establishment in Dark Web Communities

The additional layers of anonymity afforded by the Dark Web make the process of trust establishment and development even more challenging. Dark Web communities are uncertain and risky environments by default (Nurse & Bada, 2018; Yip et al., 2013), and are often frequented by users or outsiders who try to attack them, thereby undermining and endangering their existence. In order for a Dark Web forum to be successful, a balance between negative sentiment and distrust and an environment characterized by “good” behavior is required (Décary-Hétu & Dupont, 2013). More specifically, trust establishment in CSEA networks on the Dark Web is further complicated in light of the sensitive nature of the topic area. Betrayal by a trusted co-offender, or identification by law enforcement personnel who pose as a co-offender, are associated with serious risks, such as detection and exposure, which ultimately creates a structural deficit in terms of trustworthiness (Dupont et al., 2016, 2017). One may therefore argue that most ties in criminal networks online are not based on strong interpersonal relationships and social capital (as is the case in criminal networks in the physical world), but that they are sufficiently strong to provide access to sought-after resources. According to Yip et al. (2013), trust is never guaranteed and remains a vulnerable entity; it is maintained and further developed by progressing through various stages.

### Initial Identity Construction and Trust Development

In criminal networks online, and more specifically in CSEA networks on the Dark Web, a user does not tend to have an established identity in the beginning. However, in order to develop collaborative ties, and become an accepted (and eventually trusted) user, a user’s identity needs to be established over time. Open networks are therefore convenient locations for the sharing and learning of new skills, socializing, and meeting new people, as well as for the initiation of trust development to begin (Dupont, 2013). Dark Web forums, however, place emphasis on new members to demonstrate their legitimacy and reliability (Lacey & Salmon, 2015). Lusthaus (2012) suggests that forums formally or informally assess cybercriminal attributes to establish a baseline for cooperation. This may be achieved by means of (i) background checks, (ii) referrals, (iii) transcripts of previous communication, (iv) evidence of past criminal activity, and (iv) exchange of compromising information. In the same way as some of these cybercriminal forums require the provision of evidence of legitimacy and reliability, CSEA forums on the Dark Web may request CSEM for a user to join the network or to continue their membership (Broadhurst et al., 2014; Lusthaus, 2012).

This initial contact is a first step in the establishment of an online identity, which is a personal brand, and lays the foundation for a reputation that is necessary for trust to be developed further (Lusthaus, 2012; Yip et al., 2013). Reputation is one of the most important elements in being seen as a trustworthy co-offender (i.e., a precursor for trustworthiness) (Décary-Hétu & Dupont, 2013; Dupont et al., 2016). In CSEA forums on the Dark Web, members may achieve a higher status based on the quantity and quality of their contributions, which are delivered by their usernames and therefore intrinsically tied to their online identity. Members may further be rewarded for producing and sharing new CSEM (Broadhurst et al., 2014). Here, a dilemma becomes apparent—while one’s identity, reputation, and trustworthiness are associated with a username, there is a competing incentive to periodically change it in order to avoid law enforcement detection and exposure (Lusthaus, 2012; Nurse & Bada, 2018).

### Maintenance of Trust

Users who aim to establish a reputation, and sustain the cooperation with a trusted co-offender, may choose to reveal personal characteristics and engage in social and networking behaviors to support this process. Behavior deemed to be trustworthy involves portraying oneself as an active user by engaging in frequent activity, including posting messages, contributing to open discussions, exchanging valuable advice and knowledge (e.g., through tutorials), and generally being helpful, as well as mentoring and offering feedback to others (Afroz et al., 2013; Décary-Hétu & Dupont, 2013; Tzanetakis et al., 2016; Yip et al., 2013). In addition, research indicates that humor, playfulness, and sarcasm are frequently used to invoke trustworthiness (Dupont et al., 2016), and that it is the commitment and dedication users show to the community which leads to a mutual sense of belonging and trust. Social skills, such as the ability to establish and maintain a good quantity and quality of interpersonal ties, are crucial in the search for suitable co-offenders (Dupont, 2013). It is here where an in-group identity may be formed which lays the foundation for informal social control (Yip et al., 2013).

For some users, establishing a good reputation and trustworthiness may become a goal in itself, and they present with the explicit desire to achieve status by moving up the ranks in a forum (Décary-Hétu & Dupont, 2013; Lusthaus, 2012). While the primary motivation for users of CSEA forums on the Dark Web may be sexual gratification, competing for a higher status within the community is equally important in light of the associated benefits (Broadhurst et al., 2014). Trustworthiness and reputation may therefore be achieved through a combination of personal characteristics (i.e., who you are), networking characteristics (i.e., who you know), and behavioral characteristics (i.e., what you do), which need to be maintained over time, and cannot be easily feigned (Décary-Hétu & Dupont, 2013).

Once trust has been established, it must be maintained. Here, the social aspect becomes valuable—dedication to the community through engaging in frequent activity and communication (especially by sharing new and unique CSEM), unconditional cooperation, exchanging advice and knowledge, and generally being helpful and humorous, are all ways to achieve this, and are rewarded with recognition by others. Emphasis is therefore placed on a friendly atmosphere that is characterized by good behavior and politeness, appreciation, and respect toward one another (Afroz et al., 2013; Broadhurst et al., 2014; Décary-Hétu & Dupont, 2013; Tzanetakis et al., 2016; Yip et al., 2013). Long-term trust, as in the physical world, is largely related to social skills, such as repeated interaction and familiarity, and general comradeship (Cohen-Almagor, 2013; Hsu et al., 2011). Ultimately, a well-functioning network in which users get on and respect one another also make it stronger and more successful, eventually developing resilience to deterioration (Dupont, 2013). Through facilitating the formation of trusting and meaningful relationships, most users will still exchange illegal material and co-offend on CSEA forums on the Dark Web, despite the risks this involves (Cohen-Almagor, 2013; Dupont et al., 2016; Lusthaus, 2012; Yip et al., 2013).

### Limitations and Directions for Future Research

While the review has demonstrated that asking a question of the literature that combines two different perspectives is valuable for a more in-depth understanding of the topic area, a number of limitations have to be acknowledged. Naturally, studies from different disciplines vary in terms of their methods and approaches to data analysis, which impacts on the comparability across the included articles in our review. However, within each disciplinary set of included articles, studies were comparable in terms of the research questions they posed, and the methodological approaches used to address these. Nevertheless, it was noted that the qualitative approaches to data analysis were often not specified or described in the necessary detail in the criminological literature.

While both the psychological and the criminological literature lacked a focus on CSEA forums on the Dark Web, they still considered important aspects that are related to and underpin the formation of trust and relationships among users in online networks. More specifically, it became apparent that most psychological studies had been conducted with datasets that were derived from the Surface Web or samples of individuals who had been arrested for or convicted of offenses related to CSEM. None of the articles therefore specifically referred to data that had been derived from, or users that had been involved in, networks on the Dark Web. Given that most of these studies were completed between 1999 and 2015, this is perhaps to be expected. Interest in the Dark Web, and its use for illegal purposes, has received relatively little attention until 2017, when the international law enforcement operation by Taskforce Argos first offered an insight into the wide-ranging role the Dark Web played in the commission of offenses related to CSEA.

The literature would therefore benefit from a more in-depth examination of the process through which individuals seek to establish trust, and develop relationships, with other users on CSEA forums on the Dark Web. It would be of interest to explore how individuals describe this process, as well as which aspects and features they (perceive to) take into consideration when making the decision of whether or not another user is trustworthy enough to initiate contact and develop a relationship with. Further research is also needed in terms of better understanding this population from a psychological perspective. In addition, it would be useful to explore in more detail how trust-based personal relationships between co-offenders may trigger the formation of smaller sub-networks (within larger CSEA networks on the Dark Web), and how this may contribute to the progression and escalation of offending behavior. It goes without saying that the absence of such studies is at least in part due to the immense difficulty of accessing data derived from such forums.

## Conclusion

The review presented here aimed to provide an overview of the current knowledge and understanding of the nature of trust and relationship development among members of online networks that are dedicated to CSEA, both from a psychological and a criminological perspective. While the two disciplines vary in their focus, they share an interest in the topic. We were particularly interested in deriving insights from a larger literature base that may help us explain, and make better sense of, the way users on CSEA forums on the Dark Web communicate and interact with one another. The psychological literature is predominantly concerned with individuals’ motivations and the function their behavior serves, whereas the criminological literature concerns itself more with how individuals interact online. We therefore sought to answer the question of how users develop trust and relationships in a high-stakes environment (in terms of one’s identity and actions being revealed) that is predominantly used for illegal purposes, and where levels of information about others and their trustworthiness are limited.

Further contributing to our existing knowledge and understanding of this phenomenon is important in light of the implications for law enforcement and policy. Law enforcement would benefit from a more established evidence base in terms of better understanding how users operate on such networks, not only for the purpose of early detection but also in order to inform operational strategies around undercover policing. Industry in the form of public and private companies also have a vested interest in keeping up-to-date with current knowledge and understanding around the use of Internet communication platforms for illegal purposes, given their role in the monitoring of illegal content, as well as its identification and removal.

## Notes

1 It is important to highlight, however, that some users may subscribe numerous times, inflating the overall number of registrations. In addition, the number of one million user registrations may also include bots and accounts created by law enforcement personnel. The article does not clearly state whether the one million user registrations refer to unique users. Furthermore, their reference to active users raises the question of what it constitutes—it may refer to a user who has communicated and interacted on the site, or someone who has regularly logged in. Again, this is not clear from the article.

2 In Tables 3 and 4, we adopted the terminology used in each of the articles. However, we would like to point out that the use of the term “pedophile” may not be accurate in all instances in that some users may not meet the diagnostic criteria for this psychiatric disorder (as part of which an individual experiences a primary or exclusive sexual attraction to pre-pubescent children).
